# Splenic Metastases: A Fluorine-18 Fluorodeoxyglucose PET/CT Analysis of Primary Tumor Distribution

**DOI:** 10.7759/cureus.106457

**Published:** 2026-04-05

**Authors:** Aziz Gültekin, Ali Çelik, Tarık Şengöz, Fikri S Şimsek

**Affiliations:** 1 Nuclear Medicine, Pamukkale University, Denizli, TUR

**Keywords:** fdg pet/ct, metastatic disease, nonhematologic malignancies, solid tumors, splenic metastases, suvmax

## Abstract

Background and aim

Splenic metastases are uncommon and typically occur in advanced malignancies despite the spleen’s extensive vascular network. This retrospective, single-center cohort study aimed to provide an incidence-based evaluation of splenic metastases in a large PET/CT population and to assess their prognostic implications in contemporary clinical practice.

Materials and methods

Between January 2018 and December 2024, 41 cases (0.40%) of splenic metastases were identified among 10,295 patients with nonhematologic malignancies undergoing fluorodeoxyglucose (FDG) PET/CT. Metastases were verified through histopathology or, more commonly, through imaging-based criteria supported by longitudinal clinical and radiological follow-up, with consensus evaluation by two experienced nuclear medicine physicians. Maximum standardized uptake value (SUVmax) measurements were obtained for all splenic lesions.

Results

The mean SUVmax was 8.39 ± 4.03 (range: 3.89-22.22). The most common primary tumors were lung (34.15%), ovarian (21.95%), breast (9.76%), and colorectal (9.76%) cancers. All cases demonstrated concomitant metastatic involvement in other organs, with no isolated splenic metastases observed. Median overall survival was 7.1 months, with one-year and two-year survival rates of 31.7% and 28.2%, respectively. Patients with lung cancer showed significantly poorer overall survival compared to other primary tumors (HR = 2.43, 95% CI: 1.15-5.16, p = 0.020). In multivariate analysis, age and male sex were independently associated with overall survival.

Conclusions

Splenic metastases are rare (0.40%) in nonhematologic malignancies and are typically associated with widespread metastatic disease. The most common primary tumors are lung, ovarian, breast, and colorectal cancers. Survival is poor following splenic metastasis detection, with a median overall survival of 7.1 months. These findings provide valuable epidemiologic and prognostic data on splenic metastases detected by FDG PET/CT in clinical practice.

## Introduction

The spleen, despite its exceptional vascularity, represents one of the least frequently involved organs in metastatic disease from solid tumors, a phenomenon attributed to a combination of anatomical, hemodynamic, and immunological factors [1,2]. As a result, splenic metastases are often detected incidentally; their clinical significance remains underrecognized, and their prognostic implications are poorly characterized in prospective cohorts. Investigations of splenic metastasis from nonhematologic primaries have predominantly relied on retrospective autopsy series, which indicate that splenic involvement typically coexists with extensive visceral metastases [3-5]. Primary malignancies most frequently associated with splenic metastases include lung, breast, ovarian, and colorectal carcinomas, as well as melanoma [3,5-7].

The widespread adoption of fluorine-18 fluorodeoxyglucose (F-18 FDG) PET/CT over recent decades has significantly enhanced the detection and characterization of splenic metastatic lesions [8]. F-18 FDG PET/CT facilitates the metabolic differentiation between benign and malignant splenic pathologies, demonstrating preferential accumulation in malignant entities such as lymphomas and metastases [4,9]. Although data are limited, prior studies have shown that maximum standardized uptake values (SUVmax) on PET imaging exhibit substantial discriminatory capacity in identifying metastatic involvement [10,11]. However, comprehensive data on the primary tumor spectrum and metabolic characteristics of PET/CT-detected splenic metastases remain scarce, as prior studies have predominantly focused on diagnostic differentiation rather than tumor distribution and survival outcomes. To our knowledge, this study represents one of the largest PET/CT-based clinical cohorts evaluating the incidence, primary tumor distribution, and survival outcomes of splenic metastases in nonhematologic malignancies.

The present study aimed to retrospectively evaluate splenic metastases from three complementary perspectives within a large single-center PET/CT cohort: epidemiological characterization, including incidence and primary tumor distribution; metabolic assessment based on SUVmax measurements; and prognostic evaluation through survival analysis. By integrating these components, this study moves beyond purely descriptive reporting and provides a comprehensive clinical perspective on splenic metastases in the FDG PET/CT era.

## Materials and methods

Patient selection

This retrospective study included patients with nonhematologic malignancies who underwent F-18 FDG PET/CT imaging in the Department of Nuclear Medicine at Pamukkale University Hospital between January 2018 and December 2024. Nonhematologic malignancies were defined as all solid tumors and nonhematologic neoplasms, including epithelial cancers (carcinomas), mesenchymal tumors (sarcomas), melanoma, germ cell tumors, and other nonhematologic malignancies. The inclusion and exclusion criteria applied in this study are presented in Table 1.

**Table 1:** Inclusion and exclusion criteria F-18 FDG, fluorine-18 fluorodeoxyglucose

Criteria type	Criterion
Inclusion	Patients with histologically confirmed nonhematologic malignancies
	F-18 FDG PET/CT performed for initial staging, restaging, or treatment response assessment
	Incidental detection of splenic lesions with increased FDG uptake on PET/CT imaging
	Confirmation of splenic metastases through histopathologic examination, correlative imaging studies, or clinical follow-up
Exclusion	Primary hematologic malignancies (lymphoma, leukemia, multiple myeloma, and other lymphoproliferative or myeloproliferative disorders)
	Patients undergoing PET/CT for a fever of unknown origin
	Patients undergoing PET/CT for metabolic characterization

Following these criteria, 10,295 patients with nonhematologic malignancies were identified from a total of 12,275 PET/CT examinations performed during the study period. Among these, 41 patients (0.40%) demonstrated splenic metastatic involvement and constituted the study population. Verification of splenic metastases was achieved through various methods, including histopathologic examination (when available), correlative imaging studies showing characteristic progression or regression patterns, and/or longitudinal clinical follow-up demonstrating disease behavior consistent with metastatic involvement.

Ethics statement

Institutional review board approval was obtained from the Pamukkale University Non-Interventional Clinical Research Ethics Committee (approval date: December 24, 2024; decision number: 22). Written informed consent for the research use of medical information was obtained from all participants. Study conduct adhered to the principles of the Declaration of Helsinki.

Imaging protocol and analysis

Following a six-hour fasting period, patients received intravenous F-18 FDG administration (250-400 MBq; 7-11 mCi) after confirmation that fasting glucose concentrations remained below 200 mg/dL. Imaging commenced 60 minutes post-injection using a GE Discovery IQ 5-Ring PET/CT scanner (General Electric, Boston, MA, USA). Emission data acquisition proceeded at 1.5 minutes per bed position.

Low-dose CT transmission scanning employed the following parameters: 50-120 mAs, 90-140 kVp, 16-slice acquisition, and 5 mm slice thickness. Scan coverage extended from the cranial vertex to either the feet or thighs based on clinical indications. Intravenous contrast administration was not performed.

PET image attenuation correction utilized CT-derived information processed through an ordered-subset expectation maximization algorithm (33 subsets, three iterations). Image reconstruction incorporated time-of-flight technology with attenuation-corrected iterative methods. Fused PET/CT datasets were evaluated on AW Server 3.2 and AW Volume Share 7 (AW4.7) workstations (General Electric) with reconstructed transverse, sagittal, and coronal sections (5 mm thickness).

Two board-certified nuclear medicine physicians independently performed visual and quantitative assessments of F-18 FDG PET/CT studies. Consensus determination established the diagnosis of splenic metastasis. SUVmax quantification was performed on all splenic metastatic foci, with peak values recorded for analysis.

Verification of splenic metastases

Splenic metastases were diagnosed based on a combination of imaging findings and clinical follow-up. All PET/CT studies were independently evaluated by two experienced nuclear medicine physicians, and only cases with consensus agreement were included.

In the absence of routine histopathological confirmation, radiological criteria for metastasis included focal FDG uptake inconsistent with physiological distribution, corresponding structural abnormalities on CT, and/or progressive changes in lesion size or metabolic activity on follow-up imaging. Diagnoses were further supported by concordant findings across imaging modalities, consistent with real-world clinical practice.

Statistical methods

IBM SPSS Statistics for Windows, version 25.0 (released 2017; IBM Corp., Armonk, NY, USA) was used for all statistical computations. Categorical variables were expressed as frequencies and percentages. Continuous variables were presented as mean ± SD or median with range, depending on data distribution characteristics.

Overall survival was defined as the time from the first detection of splenic metastasis on F-18 FDG PET/CT to death from any cause or last follow-up. Overall survival probabilities were estimated using the Kaplan-Meier method. Median overall survival and survival rates at specific time points were calculated from Kaplan-Meier survival curves. Survival distributions between groups were compared using the log-rank test.

In addition to descriptive statistics and Kaplan-Meier survival analysis, Cox proportional hazards regression analysis was performed to evaluate potential prognostic factors associated with overall survival. Variables included age, sex, primary tumor type (lung vs others), and SUVmax values. Due to the limited sample size, a parsimonious multivariate model was constructed to avoid overfitting. HRs with 95% CIs were calculated. A p-value < 0.05 was considered statistically significant.

## Results

During the study period, 12,275 patients underwent F-18 FDG PET/CT examinations at our institution between January 2018 and December 2024. Of these, 1,980 patients were excluded due to hematologic malignancies, fever of unknown origin, metabolic characterization, or PET/CT performed for primary tumor investigation.

**Figure 1:**
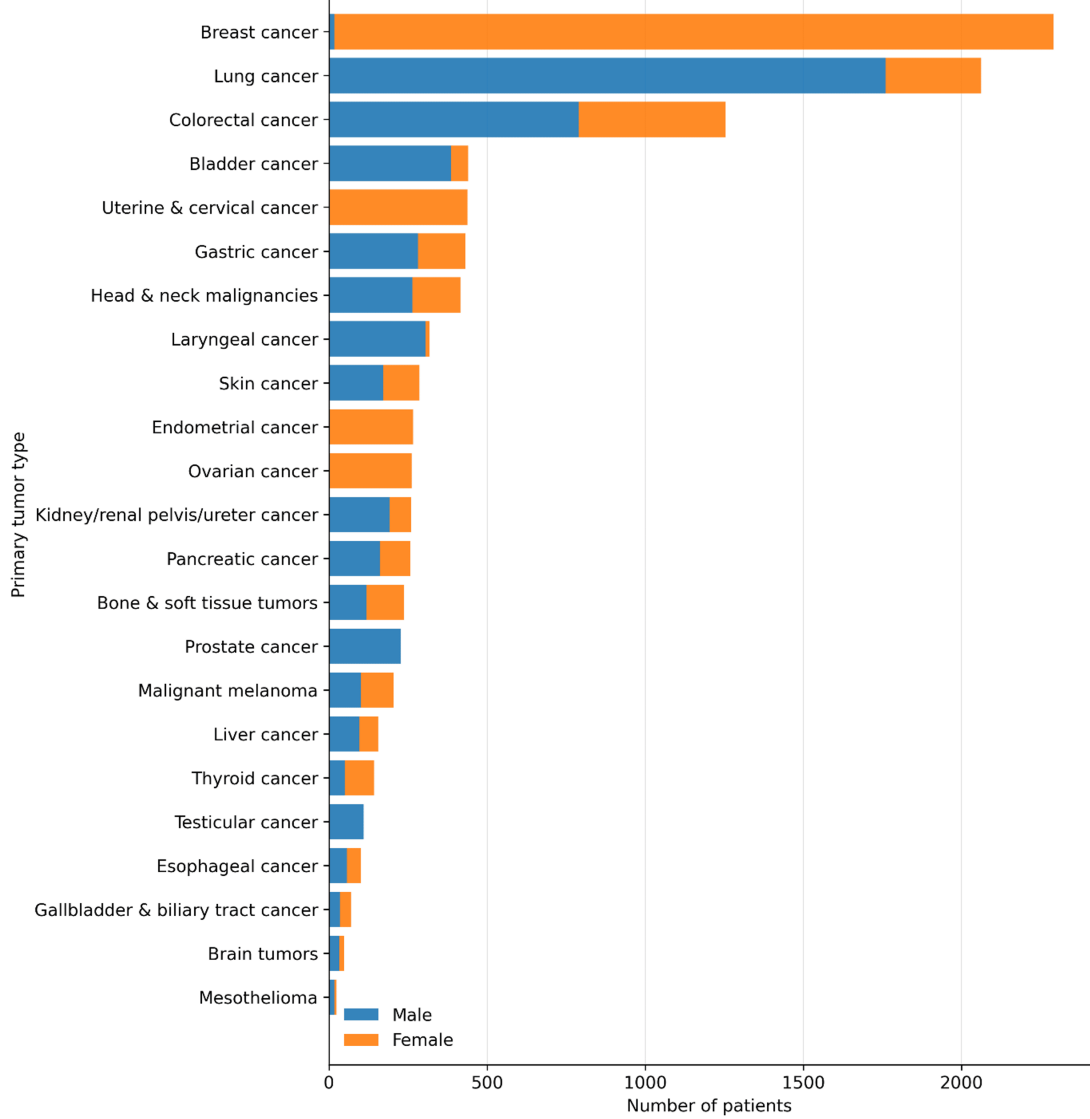
Distribution of primary tumor types in the study cohort (n = 10,295) stratified by sex Horizontal stacked bars represent the number of patients for each tumor type, with male (blue) and female (orange) proportions indicated.

Among the remaining 10,295 patients with nonhematologic malignancies, splenic metastatic involvement was detected in 41 patients on F-18 FDG PET/CT imaging (0.40%). The demographic and clinical characteristics of the overall study population are summarized in Table 2 and Figure 1. The most common primary tumors were breast cancer (22.2%), lung cancer (20.0%), and colorectal cancer (12.2%).

**Table 2:** Demographic and clinical characteristics of the study population according to primary tumor type

Primary tumor	Total, n (% of cohort)	Male, n (%)	Female, n (%)	Age (mean ± SD)	Age (min-max)
Lung cancer	2062 (20.0)	1760 (85.4)	302 (14.6)	64.22 ± 9.53	24-93
Breast cancer	2290 (22.2)	17 (0.7)	2273 (99.3)	54.40 ± 12.63	22-93
Ovarian cancer	261 (2.5)	0 (0)	261 (100)	57.53 ± 12.72	19-90
Colorectal cancer	1254 (12.2)	789 (62.9)	465 (37.1)	63.36 ± 11.81	22-94
Pancreatic cancer	257 (2.5)	161 (62.6)	96 (37.4)	63.60 ± 12.10	25-95
Endometrial cancer	266 (2.6)	0 (0)	266 (100)	62.3 ± 9.8	34-86
Bladder cancer	440 (4.3)	386 (87.7)	54 (12.3)	67.68 ± 9.65	32-91
Gastric cancer	431 (4.2)	281 (65.2)	150 (34.8)	62.27 ± 12.03	27-90
Esophageal cancer	100 (1.0)	56 (56)	44 (44)	62.52 ± 11.22	34-86
Brain tumors	48 (0.5)	33 (68.8)	15 (31.2)	59.81 ± 16.40	22-82
Thyroid cancer	142 (1.4)	50 (35.2)	92 (64.8)	56.26 ± 15.23	21-85
Testicular cancer	110 (1.1)	110 (100)	0 (0)	35.86 ± 12.86	20-81
Liver cancer	155 (1.5)	97 (62.6)	58 (37.4)	63.92 ± 11.72	27-87
Prostate cancer	227 (2.2)	227 (100)	0 (0)	69.84 ± 9.97	29-94
Gallbladder and biliary tract cancer	69 (0.7)	34 (49.3)	35 (50.7)	63.72 ± 9.65	43-88
Malignant melanoma	205 (2.0)	101 (49.3)	104 (50.7)	63.46 ± 15.06	23-96
Head and neck malignancies	415 (4.0)	263 (63.4)	152 (36.6)	59.21 ± 15.35	18-98
Laryngeal cancer	318 (3.1)	305 (95.9)	13 (4.1)	64.04 ± 9.65	20-88
Skin cancer	286 (2.8)	172 (60.1)	114 (39.9)	71.31 ± 12.22	36-98
Uterine and cervical cancer	438 (4.3)	0 (0)	438 (100)	60.1 ± 12.0	21-88
Bone and soft tissue tumors	238 (2.3)	118 (49.6)	120 (50.4)	57.11 ± 17.69	18-92
Mesothelioma	23 (0.2)	16 (69.6)	7 (30.4)	64.39 ± 16.16	24-89
Kidney, renal pelvis, and ureter cancer	260 (2.5)	192 (73.8)	68 (26.2)	62.49 ± 12.47	21-89
Overall population	10,295 (100)	5168 (50.2)	5127 (49.8)	60.93 ± 13.11	18-98

The distribution of splenic metastases according to primary tumor type is presented in Table 3. Splenic metastases were most frequently observed in lung cancer (n = 14), ovarian cancer (n = 9), breast cancer (n = 4), and colorectal cancer (n = 4).

**Table 3:** Distribution of splenic metastases according to primary tumor type

Primary tumor	Total (n)	Splenic metastasis (n)	Incidence (%)
Overall population	10,295	41	0.40
Lung cancer	2062	14	0.68
Breast cancer	2290	4	0.17
Ovarian cancer	261	9	3.45
Colorectal cancer	1254	4	0.32
Pancreatic cancer	257	2	0.78
Endometrial cancer	266	1	0.38
Bladder cancer	440	1	0.23
Gastric cancer	431	1	0.23
Esophageal cancer	100	1	1.00
Thyroid cancer	142	1	0.70
Testicular cancer	110	1	0.91
Mesothelioma	23	1	4.35
Cancer of unknown primary	88	1	1.14

**Figure 2:**
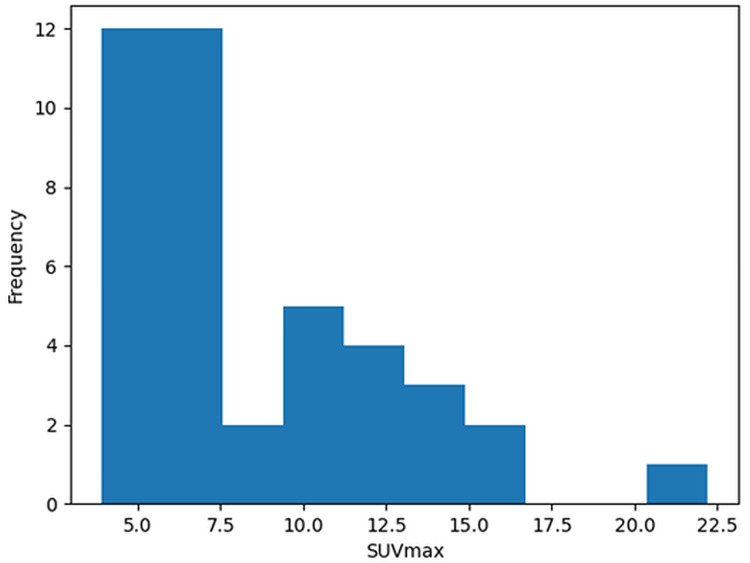
Distribution of SUVmax values in splenic metastases SUVmax, maximum standardized uptake value

Among the 41 patients with splenic metastases, the mean splenic lesion SUVmax was 8.39 ± 4.03, ranging from 3.89 to 22.22. No cases of isolated splenic metastasis were identified, and all patients demonstrated concomitant metastatic involvement in other organs. Confirmation of splenic metastases was achieved through histopathological examination or clinical follow-up with additional radiological imaging (Table 4). A histogram illustrating the distribution of SUVmax values of splenic metastatic lesions is presented in Figure 2.

**Table 4:** Confirmation methods for splenic metastases detected on F-18 FDG F-18 FDG, fluorine-18 fluorodeoxyglucose

Confirmation method	Number of patients (n)	Percentage (%)
Contrast-enhanced computed tomography	14	34.1
Follow-up F-18 FDG PET/CT	13	31.7
Histopathological examination (one biopsy and one splenectomy)	2	4.9
Clinical follow-up	11	26.8
MRI	1	2.4
Total	41	100

**Figure 3:**
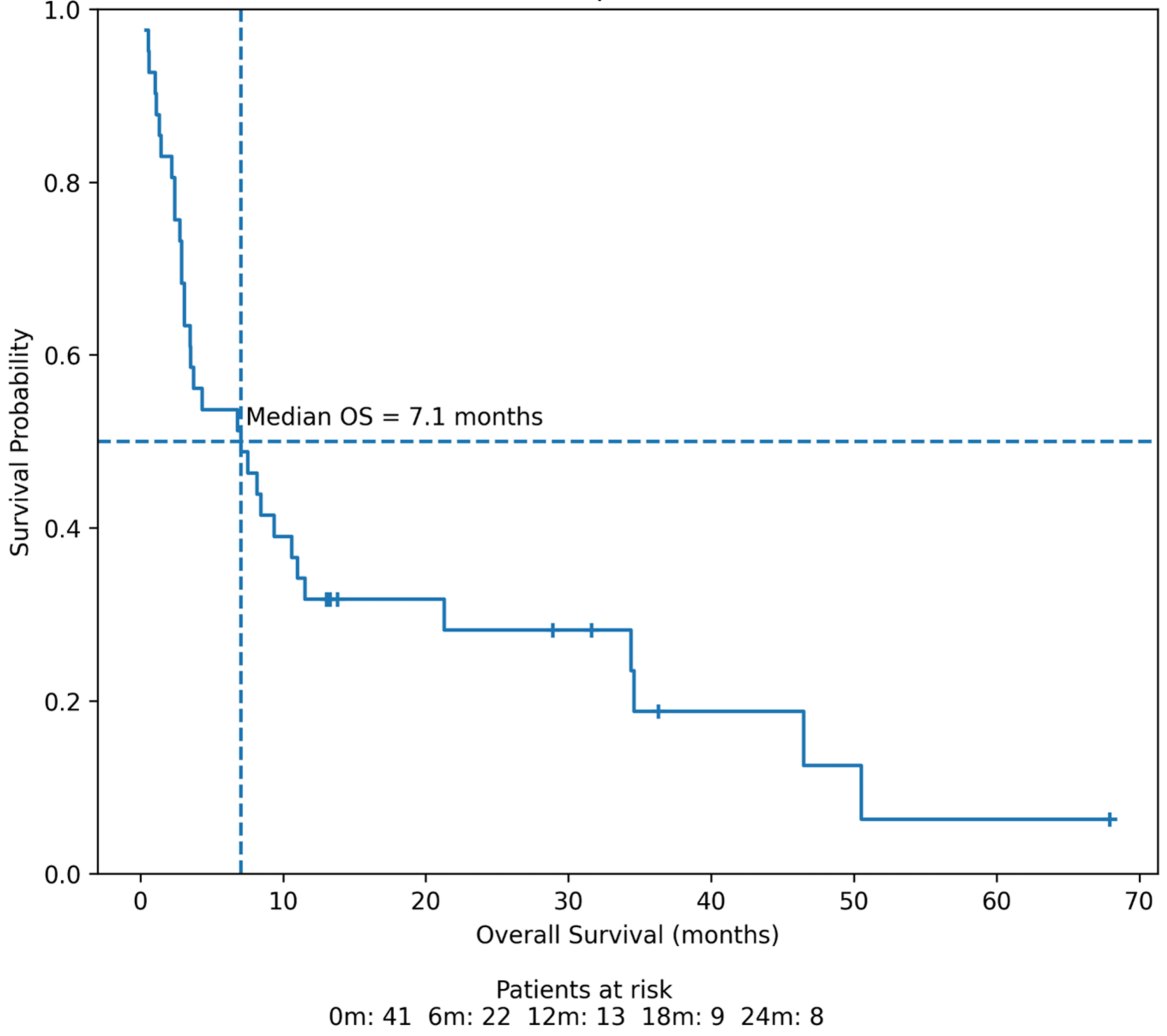
Kaplan-Meier curve demonstrating overall survival after the detection of splenic metastasis on F-18 FDG PET/CT F-18 FDG, fluorine-18 fluorodeoxyglucose

Kaplan-Meier survival analysis demonstrated poor overall survival following the detection of splenic metastasis on F-18 FDG PET/CT. The median overall survival was 7.1 months, and the one-year and two-year overall survival rates were 31.7% and 28.2%, respectively (Figure 3).

**Figure 4:**
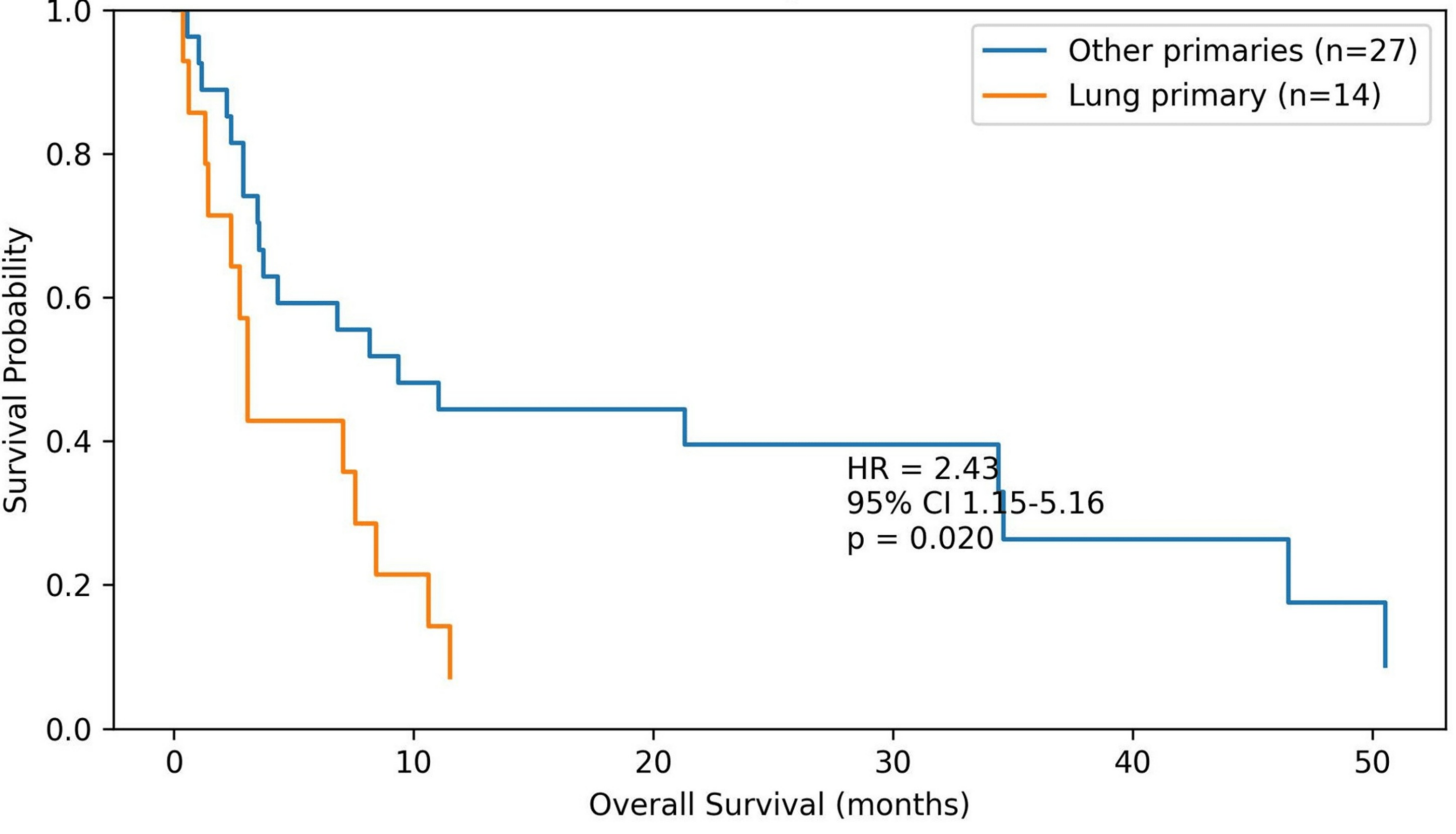
Kaplan-Meier overall survival curves comparing patients with lung primary tumors and those with other primary malignancies Patients with lung primary tumors showed significantly poorer survival (HR = 2.43, 95% CI: 1.15-5.16, p = 0.020).

Kaplan-Meier survival analysis also demonstrated significantly poorer overall survival in patients with lung primary tumors compared with those with other primary malignancies (HR = 2.43, 95% CI: 1.15-5.16, p = 0.020) (Figure 4). The numbers of patients at risk at different time points for the Kaplan-Meier overall survival curves shown in Figure 4 are presented in Table 5.

**Table 5:** Number of patients at risk at different time points for lung primary tumors and other primary malignancies Values represent the number of patients at risk at each time point corresponding to the Kaplan-Meier overall survival curves shown in Figure 4.

Time (months)	Other primaries (n = 27)	Lung primary (n = 14)
0	27	14
3	20	8
6	16	6
9	14	3
12	12	1

Among lung cancer patients with splenic metastases, five cases (35%) were classified as small cell lung cancer (SCLC) and nine cases (65%) as non-SCLC (NSCLC). Univariate Cox regression analysis demonstrated that patients with lung primary tumors had significantly poorer overall survival compared with those with other malignancies (HR = 2.43, 95% CI: 1.15-5.16, p = 0.020). Ovarian cancer was associated with more favorable survival outcomes compared with other primary tumors (HR = 0.26, 95% CI: 0.09-0.72, p = 0.014). SUVmax values were not significantly associated with overall survival, either as a continuous variable (p = 0.928) or when dichotomized based on the median value (log-rank p = 0.740).

In multivariate analysis, age and male sex remained independently associated with overall survival, whereas the effect of primary lung tumor did not retain statistical significance after adjustment. Results of the Cox regression analyses are presented in Table 6.

**Table 6:** Univariate and multivariate Cox proportional hazards regression analysis for overall survival C-index = 0.684; overall model p = 0.006 (likelihood ratio test) SUVmax, maximum standardized uptake value

Variable	Univariate HR (95% CI)	p	Multivariate HR (95% CI)	p
Age (per year)	1.05 (1.01-1.08)	0.008	1.04 (1.00-1.08)	0.035
Male sex	2.87 (1.37-5.99)	0.005	2.19 (1.01-4.77)	0.048
Lung primary	2.43 (1.15-5.16)	0.02	1.39 (0.61-3.19)	0.437
SUVmax (per unit)	1.00 (0.92-1.09)	0.924	0.96 (0.88-1.05)	0.364

## Discussion

Splenic metastases are uncommon despite the spleen’s rich vascular supply and are typically identified as part of disseminated metastatic disease [3,4]. This rarity has been attributed to several anatomical and immunological characteristics, including the acute angulation of the splenic artery, rhythmic splenic contraction, the absence of afferent lymphatic vessels, and the abundance of lymphoid tissue with potential antitumor activity [2]. Because most available data originate from autopsy series, the clinical characteristics of splenic metastases detected by modern imaging remain incompletely understood [1,3,5].

In our cohort of 10,295 patients undergoing FDG PET/CT, splenic metastases were detected in 41 patients (0.40%), consistent with previously reported clinical cohorts [6]. The true incidence may be underestimated, as lesions are often asymptomatic and incidentally detected during imaging performed for staging or follow-up [1]. Autopsy studies report a wide prevalence range (0.6-17%), likely reflecting methodological and selection differences [1,3,5].

In the present study, splenic metastases most commonly originated from lung, ovarian, breast, colorectal, and pancreatic cancers. Additional primary tumors included endometrial, bladder, gastric, thyroid, esophageal, and testicular cancers, as well as mesothelioma. One case of cancer of unknown primary was identified, for which data remain limited [12]. No isolated splenic metastases were observed, and all patients demonstrated concomitant metastatic involvement, supporting previous reports that splenic metastases typically occur in the setting of widespread systemic disease [3-5,7].

Lung cancer was the most common primary tumor, accounting for approximately one-third of cases, consistent with prior studies [1,3,6]. Although melanoma has been reported to show high splenic involvement in autopsy series [7], no such cases were observed in our cohort. Previous studies suggest a higher propensity for splenic metastasis in SCLC [13]. In our series, 35% of lung cancer cases were small cell and 65% non-small cell. A large study reported splenic metastases in 0.66% of non-SCLC patients [14], similar to our observed rate (0.68%).

FDG PET/CT plays an important role in the detection and characterization of splenic metastases by combining metabolic and anatomical information [8,10]. Conventional imaging modalities such as ultrasound, CT, and MRI may identify splenic lesions but often have limited ability to reliably distinguish benign from malignant pathology [15,16]. Because most malignant tumors demonstrate increased glucose metabolism, FDG uptake facilitates lesion detection and characterization, and previous studies have shown that FDG PET/CT can reliably differentiate benign from malignant solid splenic masses [10,17]. In our cohort, the mean SUVmax of splenic metastases was 8.39 ± 4.03, with values ranging from 3.89 to 22.22. Lee et al. reported that an SUVmax cutoff of 5.3 yielded high sensitivity and specificity for distinguishing malignant from benign splenic lesions [11], whereas other studies have suggested lower threshold values of approximately 2.2-2.3 [9]. The SUVmax values observed in our study were generally above these previously proposed thresholds. However, not all reports have shown clear metabolic separation; in a splenectomy-based series, Mainenti et al. found no significant SUVmax difference between benign and malignant lesions, although FDG PET/CT still showed a high negative predictive value [18].

Despite its diagnostic value, FDG uptake is not tumor-specific and may also be observed in infectious or inflammatory conditions [10,19]. In our study, the lack of histopathological confirmation in most cases and reliance on imaging and clinical follow-up introduce a potential risk of misclassification. Benign splenic lesions such as hamartomas, inflammatory pseudotumors, and vascular lesions, as well as infections including tuberculosis and Pneumocystis carinii, may mimic metastatic disease [7,8]. Therefore, hypermetabolic splenic lesions should be interpreted cautiously.

In selected equivocal cases, image-guided splenic biopsy may provide diagnostic confirmation, with reported success rates of 63-91%; however, in clinical practice, this procedure is often avoided due to procedural risks, particularly in patients with advanced disease and multiple metastases. Consequently, imaging-based diagnosis combined with clinical follow-up remains a commonly adopted approach [20,21]. This diagnostic approach may introduce confounding, as benign conditions can mimic metastatic involvement.

In our series, splenic metastases were consistently associated with disseminated disease, and no isolated cases were observed. Prognosis was poor, with a median overall survival of 7.1 months, consistent with previous reports [3,4]. Survival was significantly worse in patients with lung cancer in univariate analysis, reflecting the aggressive nature of this malignancy [14]. Although isolated splenic metastases may be associated with prolonged survival after splenectomy [22,23], such cases were not encountered in our cohort.

The present study extends beyond descriptive reporting by incorporating survival modeling. While lung cancer was associated with worse survival in univariate analysis, this effect did not persist after adjustment. Age and male sex were independently associated with survival, whereas SUVmax was not, suggesting that metabolic activity alone may not reflect overall tumor burden.

From a clinical perspective, the detection of splenic metastases on FDG PET/CT should alert clinicians to disseminated systemic disease, influencing staging, prognosis, and treatment decisions toward systemic rather than localized approaches. Emerging methods such as radiomics and artificial intelligence may further improve lesion characterization and prognostic stratification, although validation in larger multicenter cohorts is needed.

Limitations

This study has several limitations. The retrospective single-center design may limit generalizability, and histopathological confirmation was limited, reflecting routine clinical practice. Imaging-based diagnosis introduces potential confounding, as benign conditions may mimic metastatic involvement. Additionally, benign splenic lesions were not systematically evaluated, limiting assessment of diagnostic accuracy and SUVmax thresholds. Despite these limitations, this study provides valuable real-world data on the spectrum of primary cancers, metabolic characteristics, and survival outcomes of splenic metastases in the FDG PET/CT era.

## Conclusions

Splenic metastases are rare but represent a marker of advanced systemic malignancy. In this large FDG PET/CT cohort, splenic metastases occurred in only 0.40% of patients and were most commonly associated with lung, ovarian, breast, and colorectal cancers. All cases were associated with concomitant metastatic involvement in other organs, and overall survival after detection was poor. FDG PET/CT plays an important role in identifying splenic metastases and contributes to improved staging and prognostic assessment in oncologic imaging.

## References

[1] Giovagnoni A, Giorgi C, Goteri G (2005). Tumours of the spleen. Cancer Imaging.

[2] Chambers AF, Groom AC, MacDonald IC (2002). Dissemination and growth of cancer cells in metastatic sites. Nat Rev Cancer.

[3] Lam KY, Tang V (2000). Metastatic tumors to the spleen: a 25-year clinicopathologic study. Arch Pathol Lab Med.

[4] Schön CA, Görg C, Ramaswamy A, Barth PJ (2006). Splenic metastases in a large unselected autopsy series. Pathol Res Pract.

[5] Berge T (1974). Splenic metastases. Frequencies and patterns. Acta Pathol Microbiol Scand A.

[6] Sauer J, Sobolewski K, Dommisch K (2009). Splenic metastases—not a frequent problem, but an underestimate location of metastases: epidemiology and course. J Cancer Res Clin Oncol.

[7] Compérat E, Bardier-Dupas A, Camparo P, Capron F, Charlotte F (2007). Splenic metastases: clinicopathologic presentation, differential diagnosis, and pathogenesis. Arch Pathol Lab Med.

[8] Barat M, Hoeffel C, Aissaoui M (2021). Focal splenic lesions: imaging spectrum of diseases on CT, MRI and PET/CT. Diagn Interv Imaging.

[9] Van de Wiele C, Verstraete K, Bourgeois S, Maes A (2017). Negative 18F-FDG PET and positive CT and MRI findings in multifocal splenic hamartoma. Hell J Nucl Med.

[10] Metser U, Miller E, Kessler A (2005). Solid splenic masses: evaluation with 18F-FDG PET/CT. J Nucl Med.

[11] Lee DY, Kim YI, Ryu JS (2025). Diagnostic ability of [18F]FDG PET/CT for distinguishing benign from malignant spleen lesions. Eur Radiol.

[12] Vega EA, Yamashita S, Shin CY (2017). Laparoscopic partial splenectomy for unknown primary cancer: a stepwise approach. Ann Surg Oncol.

[13] Satoh H, Watanabe K, Ishikawa H, Yamashita YT, Ohtsuka M, Sekizawa K (2001). Splenic metastasis of lung cancer. Oncol Rep.

[14] Niu FY, Zhou Q, Yang JJ (2016). Distribution and prognosis of uncommon metastases from non-small cell lung cancer. BMC Cancer.

[15] Goerg C, Schwerk WB, Goerg K (1991). Splenic lesions: sonographic patterns, follow‑up, differential diagnosis. Eur J Radiol.

[16] Caslowitz PL, Labs JD, Fishman EK, Siegelman SS (1990). Nontraumatic focal lesions of the spleen: assessment of imaging and clinical evaluation. Comput Med Imaging Graph.

[17] Metser U, Even-Sapir E (2006). The role of 18F-FDG PET/CT in the evaluation of solid splenic masses. Semin Ultrasound CT MR.

[18] Mainenti PP, Iodice D, Cozzolino I (2012). Tomographic imaging of the spleen: the role of morphological and metabolic features in differentiating benign from malignant diseases. Clin Imaging.

[19] Karunanithi S, Sharma P, Roy SG (2014). Use of 18F-FDG PET/CT imaging for evaluation of patients with primary splenic lymphoma. Clin Nucl Med.

[20] Keogan MT, Freed KS, Paulson EK, Nelson RC, Dodd LG (1999). Imaging-guided percutaneous biopsy of focal splenic lesions: update on safety and effectiveness. AJR Am J Roentgenol.

[21] Lindgren PG, Hagberg H, Eriksson B, Glimelius B, Magnusson A, Sundström C (1985). Excision biopsy of the spleen by ultrasonic guidance. Br J Radiol.

[22] Tivadar BM, Dumitrascu T, Vasilescu C (2024). A glimpse into the role and effectiveness of splenectomy for isolated metachronous spleen metastasis of colorectal cancer origin: long-term survivals can be achieved. J Clin Med.

[23] Obana A, Komatsu N, Aiba K (2020). A case of long-term survival after splenectomy for solitary splenic metastasis from gastric cancer. World J Surg Oncol.

